# Assessing geographic and climatic variables to predict the potential distribution of the visceral leishmaniasis vector *Lutzomyia longipalpis* in the state of Espírito Santo, Brazil

**DOI:** 10.1371/journal.pone.0238198

**Published:** 2020-09-18

**Authors:** Karina Bertazo Del Carro, Gustavo Rocha Leite, Amandio Gonçalves de Oliveira Filho, Claudiney Biral dos Santos, Israel de Souza Pinto, Blima Fux, Aloísio Falqueto

**Affiliations:** 1 Tropical Medicine Unit, Federal University of Espírito Santo, Vitória, Espírito Santo, Brazil; 2 Department of Civil Engineering, Federal University of Espírito Santo, Vitória, Espírito Santo, Brazil; 3 Center for Entomology and Malacology, Espírito Santo State Health Department, Serra, Espírito Santo, Brazil; Faculty of Science, Ain Shams University (ASU), EGYPT

## Abstract

Visceral leishmaniasis (VL) is an infectious disease caused by the protozoa *Leishmania chagasi*, whose main vector in South America is *Lutzomyia longipalpis*. The disease was diagnosed in the Brazilian state of Espírito Santo (ES) for the first time in 1968. Currently, this disease has been considered endemic in 10 municipalities. Furthermore, the presence of *L*. *longipalpis* has been detected in eight other municipalities where the transmission has not been reported thus far. In this study, we performed species distribution modeling (SDM) to identify new and most likely receptive areas for VL transmission in ES. The sandflies were both actively and passively collected in various rural area of ES between 1986 and 2017. The collection points were georeferenced using a global positioning system device. Climatic data were retrieved from the WorldClim database, whereas geographic data were obtained from the National Institute for Space Research and the Integrated System of Geospatial Bases of the State of Espírito Santo. The maximum entropy algorithm was used through the MIAmaxent R package to train and test the distribution models for *L*. *longipalpis*. The major contributor to model generation was rocky outcrops, followed by temperature seasonality. The SDM predicted the expansion of the *L*. *longipalpis*-prone area in the Doce River Valley and limited the probability of expanding outside its watershed. Once the areas predicted suitable for *L*. *longipalpis* occurrence are determined, we can avoid the inefficient use of public resources in conducting canine serological surveys where the vector insect does not occur.

## Introduction

Visceral leishmaniasis (VL), also known as kala-azar, is a chronic infectious disease with high mortality if not properly treated [1]. In Latin America, the etiological agent of VL *Leishmania infantum* is transmitted by mosquitoes of the Phlebotominae subfamily, with *Lutzomyia longipalpis* (Lutz & Neiva, 1912) as the main vector [1]. In the state of Espírito Santo (ES), located in southeastern Brazil, the disease was first observed in the Doce River Valley in 1966. The first five cases of VL were diagnosed in the municipalities of Baixo Guandu and Colatina [2]. Currently, the disease affects 10 of the 78 municipalities; all the 10 municipalities are located in the central-west and northwest regions of ES [3]. However, the presence of *L*. *longipalpis* has been detected in eight other municipalities where the transmission has not been reported thus far [4]. These regions are characterized by sloped landscapes, warm weather, and low precipitation [3, 5].

The environment affects the composition, distribution, and behavior of the vectors and reservoirs involved in the transmission of VL, which impacts the epidemiology of the disease [6]. Therefore, since the transmission cycle of VL depends on an insect vector, the environmental characteristics that favor its occurrence must be investigated and identified.

In Brazil, with respect to canines as a potential reservoir of the disease, the frequent movement of people implies a lack of control in the transport of infected dogs from one region to another, resulting in an improper use of resources for investigating canine disease in areas where the VL vector does not exist [7]. Hence, the presence of the vector must be investigated before performing any other actions on the canine reservoir.

Species distribution modeling (SDM) is a technique that uses machine learning algorithms to predict the distribution of a species across geographic space and time [8]. This approach identifies regions containing suitable environmental conditions based on habitat characteristics at locations of known species occurrences [9]. Hence, the use of these tools to identify suitable areas for a disease vector occurrence would be instrumental in directing efforts toward regions that truly need it. SDMs can reveal areas with underestimated vector populations where disease transmission is unknown but can potentially occur [10–12].

Considering that the transmission of VL is through an insect vector, whose frequency and distribution are affected by environmental variables, geographic information coupled with SDM tools can be used to identify factors associated with the presence of *L*. *longipalpis* in ES. Therefore, predictive spatial modeling can help identify new and potential receptive areas for the transmission of VL in ES.

## Materials and methods

### Study area

ES is located on the Atlantic coast of southeastern Brazil, between parallels 17°53’29″–21°18’03″S and meridians 39°41’18″–41°52’45″W. It has an area of approximately 46,000 km^2^, with a maximum north-south extension of 374 km and a width of 130–150 km [13]. ES accounts for 0.5% of the Brazilian territory and comprises 78 municipalities.

The ES territory is longitudinally intersected by the Serra do Mar, a 1,500-km long system of mountain ranges and escarpments. The elevation reaches up to 2,900 m above sea level in Pico da Bandeira, Serra do Caparaó. It has a tropical climate, with an average annual mean temperature of 22 °C (9–24 °C) and average annual precipitation of 1,200 mm (900–1,600 mm) [14]. It also has extensive flat areas along its coast, with a wide variety of ecosystems determined by their natural geographical features [13].

The state encompasses the southern part of the Central Corridor of the Atlantic Forest, which comprises one of the primary areas of ombrophilous dense forest within this biome. There is a high level of endemism and species diversity in this region, which continues to be threatened by human activities [15].

### Entomological data

Phlebotomine occurrence data were obtained from samples collected from several localities in rural areas of the 78 municipalities of ES between 1986 and 2017. The phlebotomines were collected in the first three hours after sunset by active searching with a manual suction tube (Castro collector) and by passive capture using light-based traps. The active search for sandflies was performed within 30 m from the human houses, collecting resting insects on the inner and outer walls of the houses and their annexes, animal shelters, tree trunks, and Shannon traps. Passive collections were performed simultaneously using the Centers for Disease Control and Prevention traps installed in wild areas. Experienced technicians performed the sampling, regardless of the occurrence of any disease outbreak. The sampled specimens were identified according to the identification keys reported by Galati (2003) and Young and Duncan (1994) [16, 17]. All sampled localities were georeferenced using a global positioning system device.

The sampled localities where *L*. *longipalpis* was not found were considered absence points for one of the model evaluation techniques.

To reduce the sampling bias of the presence and absence occurrence points of *L*. *longipalpis*, we spatially rarefied the occurrence data using SDM Toolbox v2.4 extension for ArcGis. This tool removes spatially autocorrelated occurrence points by reducing multiple occurrence records to a single record within a specified distance.

### Environmental variables

The environmental variables used for the modeling included 19 bioclimatic variables and three topographic variables (elevation, terrain slope, and rocky outcrops).

The 19 bioclimatic variables, derived from temperature and precipitation, were obtained from the WorldClim database (version 2.1, released in January 2020, https://www.worldclim.org/). The climatic data for the creation of these bioclimatic variables were generated through interpolations of climate data obtained from approximately 50,000 weather stations distributed worldwide from 1970 to 2000 [14].

The topographic variables—elevation and terrain slope—were retrieved from the Brazilian Geomorphometric Database (TOPODATA, *Banco de Dados Geomorfométricos do Brasil*, http://www.dsr.inpe.br/topodata/) of the National Institute for Space Research (INPE, *Instituto Nacional de Pesquisas Espaciais*). Data from the Shuttle Radar Topography Mission from the US Geological Survey were used to generate these two topographic variables.

The variable rocky outcrops was acquired from the Integrated System of Geospatial Bases of the State of Espírito Santo (GEOBASES, *Sistema Integrado de Bases Geoespaciais do Estado do Espírito Santo*, https://geobases.es.gov.br/). Mapping of the rocky outcrops was performed on an orthomosaic using photointerpretation analysis and manual vectoring procedures. For better applicability in this study, this variable was transformed from categorical to continuous using the kernel density estimation with a bandwidth of 0.1°.

All variables were raster datasets, with a resolution of 30 arc seconds, which is approximately equivalent to 1 km.

The Geographic Information System ArcGIS version 10.8 (ESRI, Redlands, CA, USA) with the World Geodetic System (WGS84) datum were used for the spatial procedures of this study.

### Species distribution modeling

SDM uses machine learning computer algorithms to predict the distribution of a species using its known occurrence data and environmental variables [18]. Maximum entropy fitting is a general-purpose machine learning approach, which uses a mathematical formulation for modeling species distributions with presence-only data [18, 19]. This algorithm is based on the principle of maximum entropy, a method used to choose the best probability distribution that fits the study data [18, 20].

We used the MIAmaxent R package v1.1.1, running under R version 4.0.0, for the modeling procedures of this study. MIAmaxent is a presence-only modeling approach that provides a statistical method to model species distributions similar to Maxent’s, but it has subset selection instead of LASSO regularization. The simpler models typically produced by subset selection are ecologically more interpretable, making distribution models more grounded in ecological theory [21]. The package also executes a variable transformation based on expected occurrence–environment relationships [22].

We split the occurrence data into training and test sets for model evaluation. The final model, however, was trained using all the occurrence records and replicated 100 times, randomizing only the background points.

### Model evaluation

For model evaluation, we used two types of train-test splits—(1) random cross-validation, which splits the data randomly into 75% for training the model and 25% for testing the model, and (2) spatially stratified cross-validation, which partitions data according to the latitude and longitude lines that divide the occurrence localities into four bins of equal numbers. Both occurrence and background localities are assigned to each of the four bins based on their position concerning these lines.

Spatially stratified cross-validation is an alternative method of dealing with spatial autocorrelation, which is very likely to inflate performance in random cross-validation [23].

To account for the out-of-sample prediction error in the random cross-validation, we replicated the process 100 times. Spatially stratified cross-validation was performed using the “block” method, available in the ENMeval R package, that conducts spatially independent evaluations and estimates optimal model complexity for SDM [24].

Each of the two train-test splits described above was applied for model evaluation using random background points as species pseudo-absence and using real localities defined as species absence.

Then, we used the receiver operating characteristic (ROC) plot method. An ROC plot is created by plotting the sensitivity values (the true-positive fraction) against the 1 –specificity (the false-positive fraction) for all available probability thresholds [25]. The area under the curve (AUC) derived from the ROC plot can be interpreted as a measure of the ability of the algorithm to discriminate between a suitable environmental condition and a random analysis pixel [18]. The closer the AUC value is to 1.0, the better the performance of the model. For this study, we used the interpretation by Hosmer and Lemeshow (2000), which considers AUC values of 0.5–0.6 (no discrimination), 0.6–0.7 (discrimination), 0.7–0.8 (acceptable model), 0.8–0.9 (excellent model), and 0.9–1.0 (outstanding discrimination) [26].

To minimize problems with this measure, we constrained our analysis to a well-studied region, averaged the AUC from several replicates of the original presence data, and used the real absence data [27, 28].

The predictive contribution of each variable for the final model was obtained using the fraction of total variation explained [29]. This is represented by the increase in the fraction of deviance explained when a given variable is added to the model [12].

## Results

**Fig 1:**
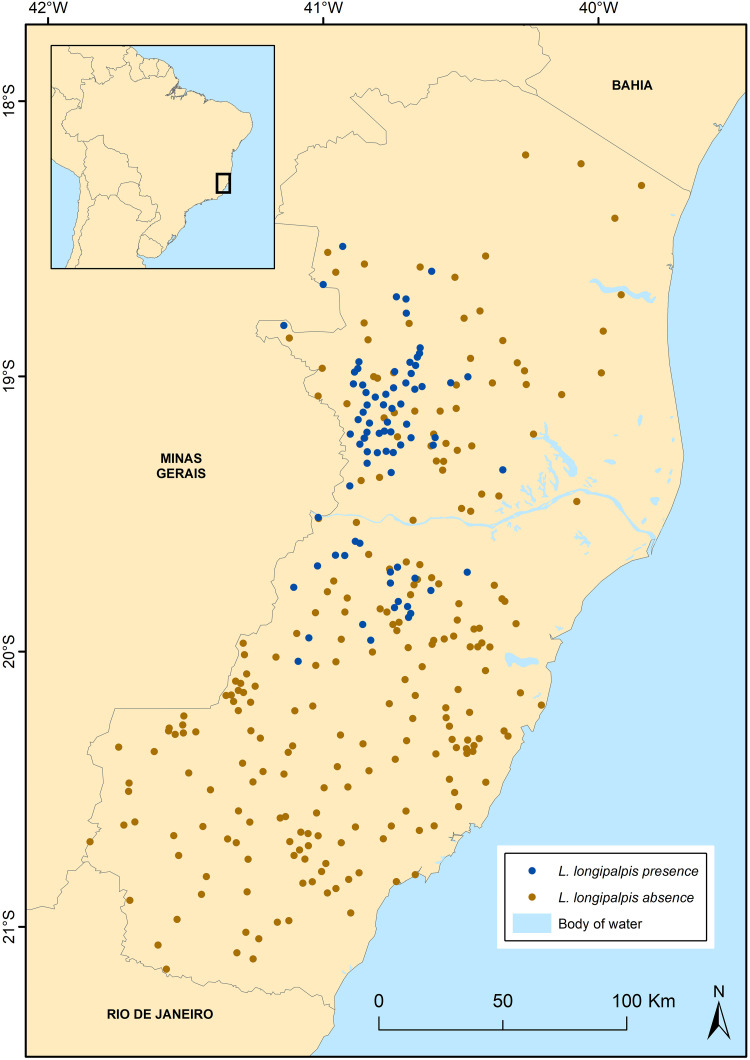
Sampling localities of phlebotomine sand flies in the state of Espírito Santo, southeastern Brazil, during the period between 1986 and 2017. Green dots represent the localities where *L*. *longipalpis* was collected, and red dots the localities where other phlebotomine species were collected but not *L*. *longipalpis*.

During the three decades of sandfly collection in ES, more than 13,600 specimens of *L*. *longipalpis* were collected from 82 unique localities throughout the state. Although the sampling effort covered the entire state, the species were only recorded in 18 of the 78 municipalities. These collection data revealed 78 presence and 262 absence points for *L*. *longipalpis* after the application of the spatially rarefying method (Fig 1).

**Fig 2:**
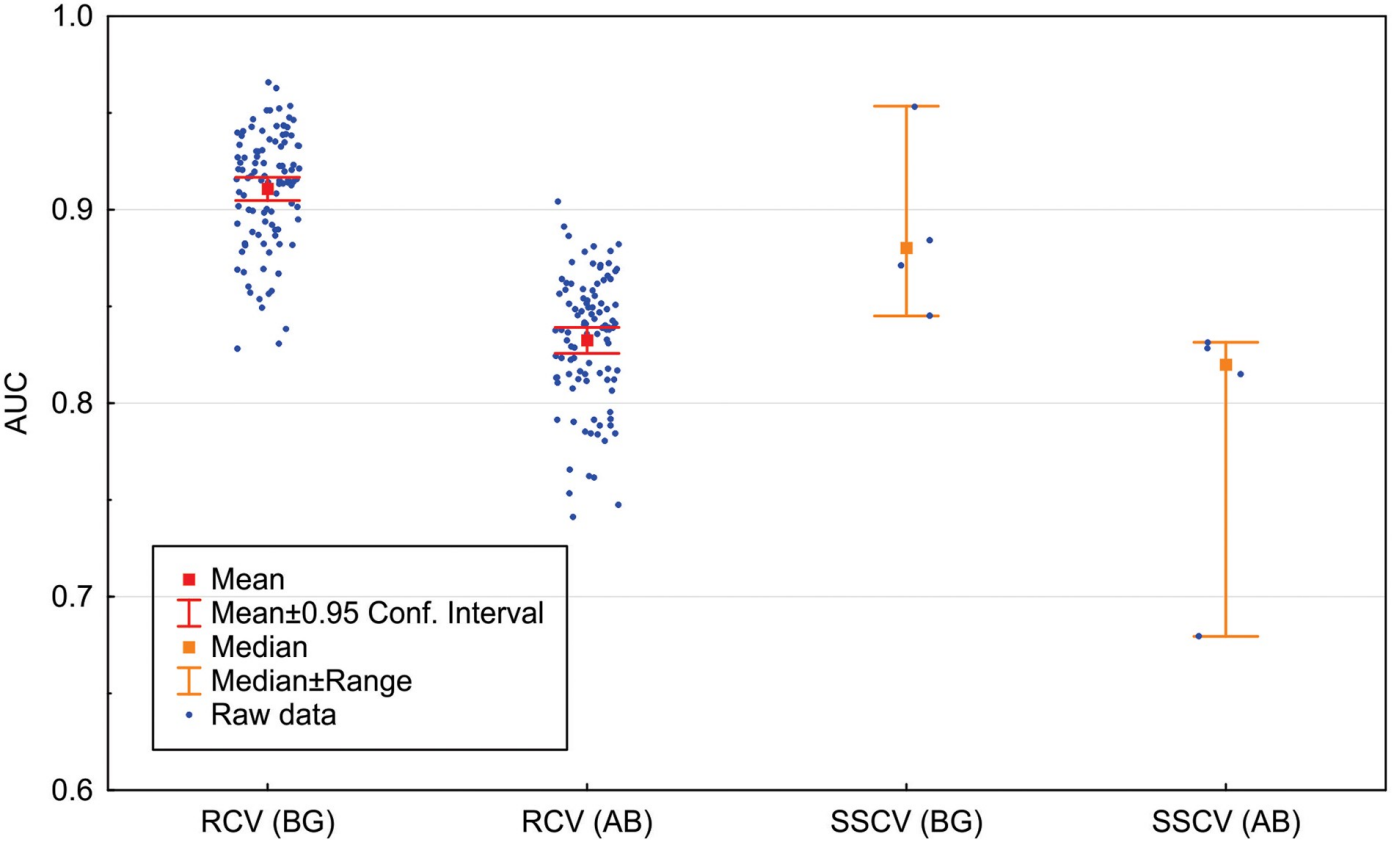
AUC model validation results, including the random cross-validation (RCV) and the spatially-stratified cross-validation (SCV), both using random background points (BG) and absence points (AB).

The model evaluation for (1) random cross-validation using background points revealed a mean AUC of 0.91 (n = 100; IC95 = 0.006); (2) random cross-validation using the species absences revealed a mean AUC of 0.83 (n = 100; IC95 = 0.007); (3) spatially stratified cross-validation using background points revealed a median AUC of 0.88 (n = 4; range = 0.85–0.95); and (4) spatially stratified cross-validation using the species absences revealed a median AUC of 0.82 (n = 4; range = 0.68–0.83). We used median and range for the spatially stratified cross-validation AUC representation because they are more appropriate measures to deal with only four bins of the block method area stratification (Fig 2).

The fraction of total variation explained, used for accounting the contribution of each variable in the final model, indicated the following variable contributions: (1) a rocky outcrop density mean of 66.79% (n = 100; 95% confidence interval [CI] = 0.41); a temperature seasonality (BIO4) mean of 19.97% (n = 100; 95% CI = 0.31), a temperature annual range (BIO7) mean of 8.38% (n = 99; 95% CI = 0.31); and a precipitation of the wettest quarter (BIO16) mean of 4.06% (n = 72; 95% CI = 0.18).

**Fig 3:**
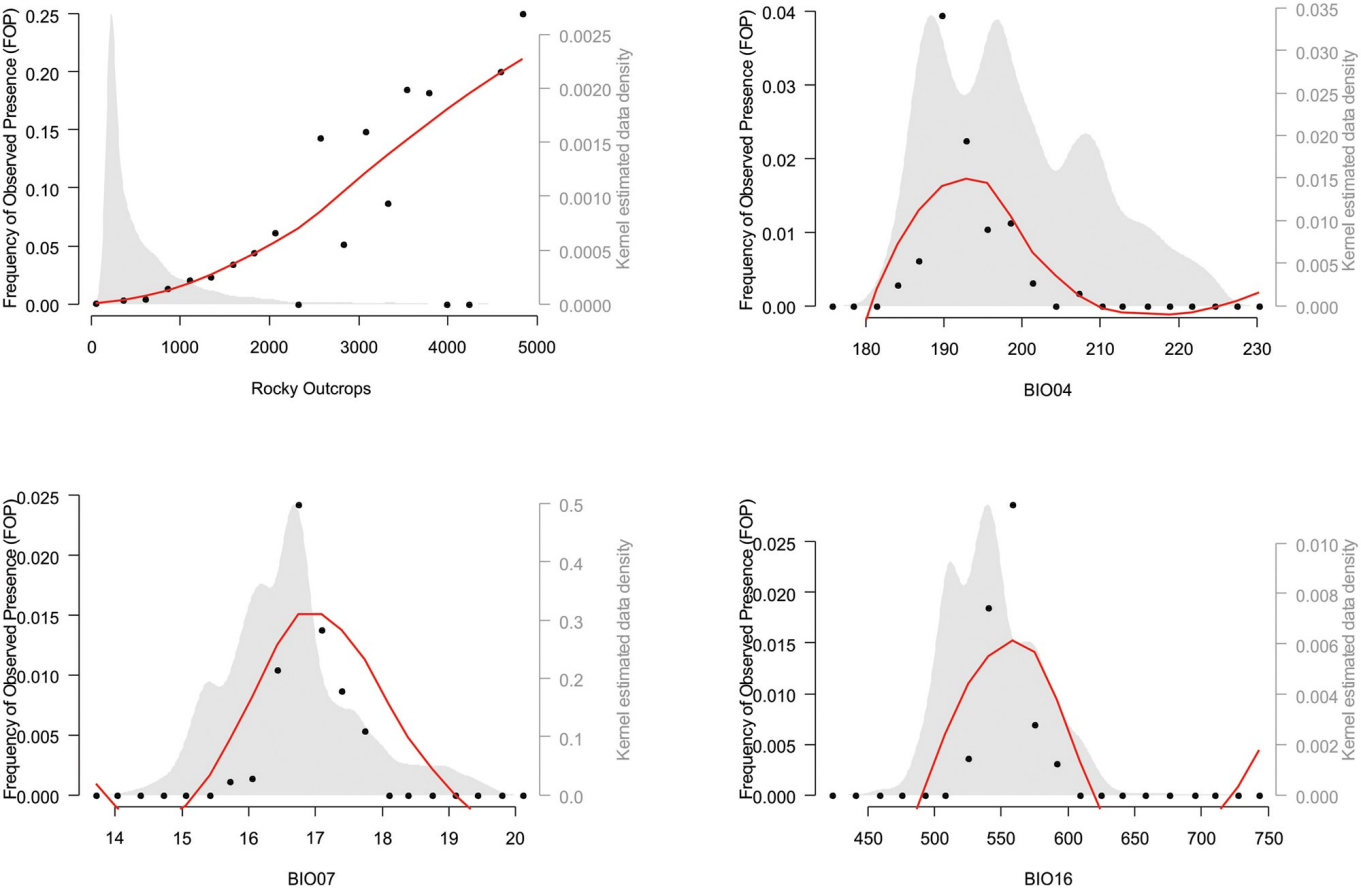
Frequency of observed presence of *Lutzomyia longipalpis* represented by the black dots and the red line as a local regression smoother across the significant environmental variables retained in the final model: Rocky outcrops, temperature seasonality (BIO04), temperature annual range (BIO07), and precipitation of the wettest quarter (BIO16).

The frequency of the observed presence plot shows how commonly *L*. *longipalpis* occurs across the range of the environmental variables kept in the final model, making it possible to recognize patterns in the observed frequency of occurrence (Fig 3).

**Fig 4:**
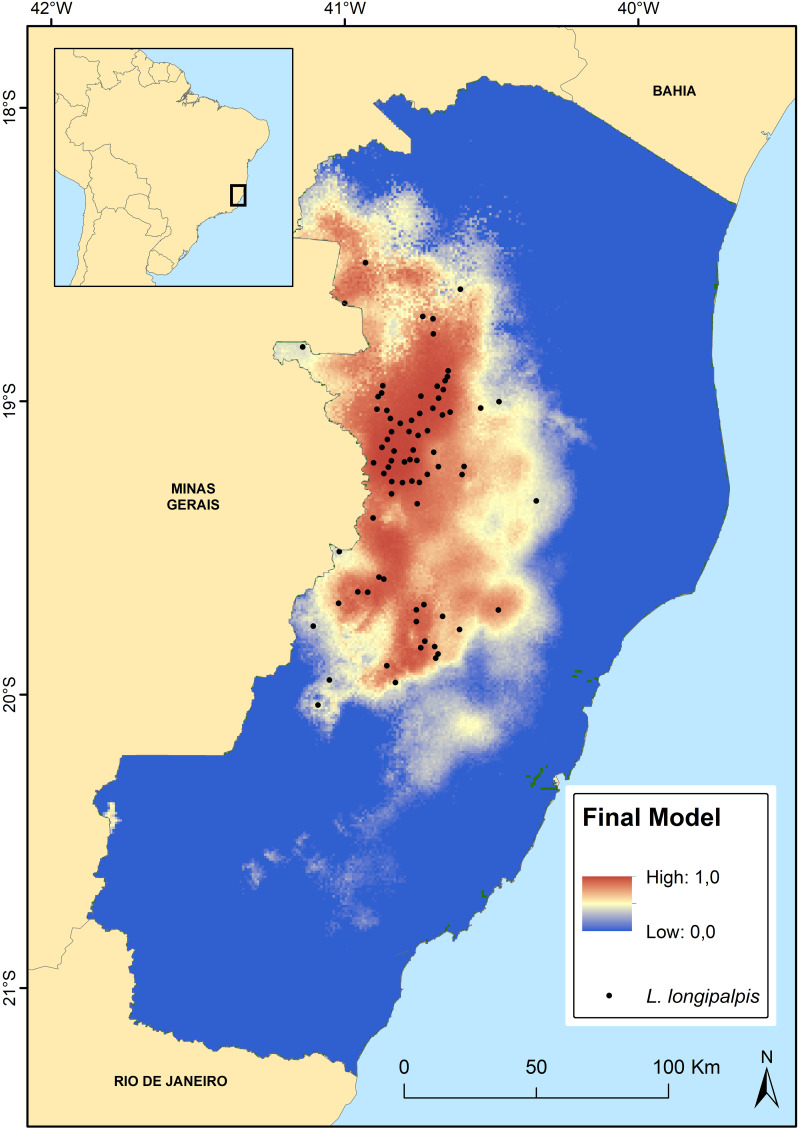
Final model normalized predicted distribution for *Lutzomyia longipalpis* in the state of Espírito Santo, southeastern Brazil. The occurrence points used in the modeling are also shown on the map.

The final model projected on the map indicates areas predicted suitable for *L*. *longipalpis* throughout ES. The predicted areas are consistent with the current knowledge regarding species distribution. However, seven municipalities—Marilândia, São Domingos do Norte, Alto Rio Novo, Barra de São Francisco, Ecoporanga, Vila Valério, and Linhares—where the species was never found in several years of entomological studies showed areas predicted to be suitable for its occurrence (Fig 4).

## Discussion

This study defines the areas predicted to be suitable for the occurrence of *L*. *longipalpis*, the VL vector, in the state of ES, suggesting possible areas of environmental risk for the transmission of the disease to both dogs and humans. This finding can be used to help public health agencies identify areas conducive to vector development, enabling their serological and entomological research efforts specifically focused on these regions rather than throughout the state.

The number of studies assessing occurrence of leishmaniasis using SDM is increasing, both in Brazil and abroad. These studies, in addition to covering all links in the disease cycle, such as vectors, reservoirs (dogs), and human diseases, included climate change scenarios [30–35].

In Brazil, the municipality of Itapira, located in the state of São Paulo (SP), was the focus of an SDM study for predicting a risk area for the spread of cutaneous leishmaniasis [36], whereas environmental niches for VL were studied in the state of Bahia (BA) [37]. VL risk areas were also investigated in the Brazilian states of Piauí [38], Mato Grosso do Sul [39, 40], Minas Gerais (MG) [41], and Rio de Janeiro (RJ) [42]. In ES, Meneguzzi et al. (2016), using SDM, investigated the association between cutaneous leishmaniasis cases and five sandfly vectors, with *Lutzomyia intermedia* considered the main vector in the state for its strongest associations with disease cases [43].

In this study, we used a modeling protocol that is at the forefront of current methods. The MIAmaxent package modeling process deals with several known flaws of SDM such as variable transformation and model selection [22]. We applied the best practices in our modeling effort, including accounting for sampling bias/spatial autocorrelation rarefying the species occurrence, setting a suitable study area extent, averaging the AUC from several replicates to account for the out-of-sample performance of the model, and using spatially stratified cross-validation and species absence for model validation [27, 28, 44]. The four distinct types of AUC validations were applied to our models to compare the extremes—at one extreme was the most widely used random cross-validation with background points and to the other extreme was spatially stratified cross-validation with species absence. The former is known to inflate the AUC, and the latter is known to provide a more reliable and realistic AUC value. Even the relatively rigorous methods for model validation resulted in a median of 0.82, which according to Hosmer and Lemeshow (2000) classifies it as an excellent model.

The occurrence region of VL in ES spans across the Doce River Valley. Furthermore, SDM for *L*. *longipalpis* showed that the environmental suitability areas match the VL occurrence areas, revealing that most of the state does not have favorable geoclimatic conditions for vector development.

This study emphasizes the planning of VL-control actions by restricting entomological and canine serological surveys to areas predicted to be suitable for vector occurrence, thereby saving financial resources and increasing the probability of finding more receptive sites for the disease. For example, the Brazilian Ministry of Health has recently decided to conduct a serological survey of 10% of the dog population of the entire state, which is an expensive affair and could be deemed unnecessary by our study.

According to our prediction, the coastal region of ES is an unlikely region for the occurrence of *L*. *longipalpis*, as observed in BA, where the vector was observed in Caatinga [37]. However, in RJ, where climatic and geographical conditions were similar to those of ES, this insect has been found in 17 municipalities thus far, including those near coastal areas where the precipitation is higher [45–49].

A possible explanation is that several studies identified *L*. *longipalpis* as a polymorphic species i.e., a complex of species [50–57]. Additionally, the genetic variant found in RJ, 9MGB ([S]-9-methyl-germacrene-B), differs from the variant that is found in ES (cembrene-1) [58]. Thus, the ecological needs of these two genetic variants may differ, helping them adapt to different environmental conditions [59, 60]. Hence, other *L*. *longipalpis* genetic variants may eventually occupy previously unoccupied areas of ES, like they occurred in the locality of Cemitério do Caju, RJ by the transport of soil in plant pots from regions where the species occurred [61]. The states of Ceará and MG, for example, have records of these two morphospecies occurring in sympatry. Additionally, the states of MG, SP, and Tocantins recorded the occurrence of more genetic variants in their territories, three in the first and two in the last two states [58].

In 2017 and 2018, phlebotomine collections were carried out and the SDM was validated by confirming the presence of *L*. *longipalpis* in new locations in three municipalities of ES—Barra de São Francisco, Nova Venécia, and Santa Teresa (personal communication).

In our study, the climatic variable that was most associated with the occurrence of *L*. *longipalpis* was rocky outcrops. This finding corroborates the theory formulated by us during several years of work in ES. The preliminary arbitrary *L*. *longipalpis* collections were carried out primarily in areas with hot and dry climates, in elevations not exceeding 500 m, and with the presence of rocky outcrops. In this context, the northwest region of ES, which is the intermediate portion of the Doce River basin, appears to be studded with rocks that dominate the local landscape.

In the southern region of ES, the locality of Estrela do Norte (20°34’48”S and 41°19’1.99”W) in the municipality of Castelo represents another area with geographic and climatic characteristics similar to those found in the Doce River basin. This geographical area, located in the Itapemirim River watershed, exhibits an important cluster of rocky outcrops. However, although this region seems to be adequate for the environmental needs of *L*. *longipalpis*, no specimen has been found there thus far. The absence of the LV vector in this area could be explained by the fact that the municipality of Castelo is geographically isolated from the Doce River Valley by a mountain range, which is impossible to be transposed naturally by the insect. The mountainous region that separates the two main hydrographic basins of ES has elevations above 1,000 meters, presenting unsuitable climatic characteristics for the occurrence of the species.

We believe that the colonization of *L*. *longipalpis* could occur in the municipality of Castelo, ES. This process could occur by mechanical transport of soil or agricultural plant seedlings from one region to another; however, there is currently no evidence of such transit between the regions. It is also plausible to think that in the Doce River Valley, the insect was introduced in ES by the transportation of cargo from the neighboring state of MG, where LV has been detected since 1959 [62].

A theory to explain the substantial relevance of rocky outcrops in predicting the occurrence of *L*. *longipalpis* would be that during the day, the rocks accumulate a great amount of heat. The slow dissipation of heat during the night would allow minor oscillations in the local temperature, generating favorable conditions for the occurrence of the vector. In contrast, in more elevated regions of ES, where there are also some rocky outcrops and the species does not occur, the thermal oscillation would be greater since temperature varies inversely to the elevation.

Temperature seasonality was the second most influential variable in the model. In this study, we noticed that in ES, *L*. *longipalpis* were predominantly observed in regions with a hot climate and a small temperature variability throughout the year. These areas have low elevations, corroborating the influence of temperature. In ES, the LV vector is observed in regions with elevations not exceeding 500 m.

In other Brazilian regions, *L*. *longipalpis* occurrence is associated with elevations lower than 900 m. In Belo Horizonte, MG, Margonari et al. (2006) [63] found a positive, albeit nonsignificant, association between VL and elevations up to 880 m, whereas Saraiva et al. (2011) [64] noted that both canine and human cases of VL were concentrated at elevations from 750 to 850 m. Conversely, in the state of São Paulo, VL vectors and hosts preferentially occurred at elevations from 274 to 539 m [65].

Most of the Doce River watershed is located in MG, the ES bordering state. There are many areas in these states sharing similar geographic and climatic conditions, mainly those located in the Atlantic Forest biome. Thus, we believe that our findings can be extrapolated to these neighboring states.

In this context, SDM enhances knowledge and provides a path to researchers and the government toward allocating human and financial resources to areas where they are significantly needed. Additionally, once the potential areas for *L*. *longipalpis* occurrence are determined, we can avoid the inefficient use of public resources in conducting canine serological surveys where the vector insect does not occur.
